# The past, present and future of Scientific discourse

**DOI:** 10.1186/1758-2946-3-46

**Published:** 2011-10-14

**Authors:** Henry S Rzepa

**Affiliations:** 1 Department of Chemistry, Imperial College London, Exhibition Road Campus, London SW7 2AZ, UK

## Abstract

The science journal is 346 years old in 2011, having evolved continuously but largely incrementally over that period. Its reinvention for an online presence has largely preserved its previously printed nature, in the sense that much of the increased functionality which is potentially offered by this new medium has yet to be exploited. In the present article an attempt is made to discuss two previously published papers, one in 1953 and the other in 2010, and to illustrate how additional functionality can be implemented in the form of accessible data sourced from quantum mechanical calculation and how subsequent discourse in the form of blogs may add to the process. In this sense, the reader of this article is invited to try for themselves whether these enhancements improve their scientific understanding, and whether such enhanced journals are good models for the future evolution of the genre.

## Introduction

The first journal devoted exclusively to science is generally accepted to have first appeared in 1665 as the Philosophical Transactions (of the Royal Society). The inaugural issue [1], which famously carries an account by Robert Boyle of "a very odd monstrous calf", is perhaps not science as we know it nowadays, but it does remind one rather of what one might find in a personal blog, a similarity I will return to later. The structure of the scientific journal and the articles published by this means evolved constantly and mostly incrementally during the next 330 years. One of the more significant, but nevertheless still incremental changes was adding an online presence during the late 1990s. Indeed, some journals founded in the late 1990s offered only an online version [2] and there are signs that some older journals may be preparing to abandon the (relatively expensive) printed form. Access to the online versions in 2011 is predominantly *via *a format which is perhaps best described as digital paper (PDF), although most journals also offer the articles in an alternative hypertext (HTML) format. There is no journal yet that has adopted a format increasingly used for books, the epub/epub3 standard [3]. On the horizon is also the fifth major evolution of hypertext markup language known as HTML5 [4,5], which strives to offer a richer interactive medium to the reader. Certainly machines (*e.g*. such as those working on behalf of search engines such as Google) now also automatically process the articles published in the modern journal, indexing the full-textual content, adding rich metadata on the topics therein described, and noting (but largely incapable of truly indexing the context of) the images and figures. Software can also usefully replace the old processes of binding the printed journal and the storage of volumes on shelves in a library or an office, a process delightfully described (by biologists, not chemists) as *defrosting the digital library*[6]. These processes largely address only the bibliographic issues (*via *rich metadata harvesting) rather than attempting to defrost the scientific or chemical content itself. It is the issues involved in defrosting the latter type of information and data that the present article addresses.

Data has always been the "elephant in the room" of scientific publishing. Because the costs of printing and distributing paper are still significant to this day, print was never really been considered a viable mechanism for distributing the (often very large amounts of) data, whether raw, or partially processed, on which almost all scientific models, theories and their interpretations are based. Instead, starting in the early 1990s and coincident with the first introduction of the Internet, many science journals offered an annex to the main journal in the form of supporting or supplemental information. This was provided in final form by the authors themselves, and the journal itself added little extra value such as indexing to this (often purely visual) content. It was very much up to an interested reader to add their own value to any (visual, textual or numerical) supporting data that might be associated with an article.

The long view over a 350 year period is that these evolutions of the journal could be regarded as largely relating to the production and delivery processes of journals, and arguably have not been matched by similar advances in how scientists *consume *or use journals. In this essay, I will analyze two chemical articles, published in respectively 1953 and 2010, from the point of view of how the original journal presented the scientific discourse, what the limitations of that presentation might have been, and the prospects of how it could evolve into a step-change rather than incremental change in that discourse.

## The relationship between a journal article and data

**Figure 1:**
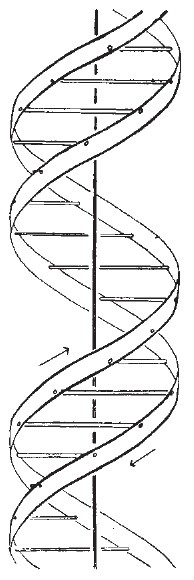
**The DNA double helix (reproduced with permission **[8]**), showing a right handed or B-helix**.

I start the analysis with article that contains (*inter alia*) what has been described as the most famous scientific diagram of the 20th Century, the representation [7] of the double helical structure of the DNA molecule by Watson and Crick (Figure 1).

**Figure 2:**
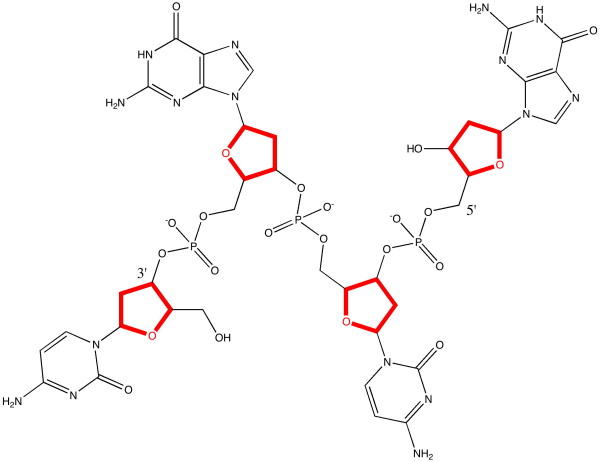
**The molecular basis of one strand of DNA, based on the CG bases**.

Indeed, this diagram is the **only **one that actually appears in the article, and one would seek in vain any diagrammatic elaboration of what the molecular structure of DNA is (although components such as deoxyribose or guanine are named as such in the text). Anyone seeking to repeat Watson and Crick's model building would certainly have to acquire additional molecular data from another sources. Some of that missing information is shown here in Figure 2, although this only describes the connectivity of the various atoms in a single strand of DNA, and not the two or three dimensional relationships of the (125 in this example) individual atoms. Note also that this diagram is presented here for visual consumption by a human, who still has to recover additional semantics such as the stereochemistry at the three stereogenic ribose centres, and note carefully that the unit represented must be accompanied by positively charged counter-ions.

Armed only with the one diagram actually published, curiosity might lead one to pose a scientific question such as "How did Watson and Crick assign the helix as right rather than left handed"? In other words, on what data did they base that conclusion? This does matter! For example, some 733 articles have appeared in the science literature over the last 20 years or so where DNA is represented as having left-handed helicity, in most cases certainly erroneously [8]. Coincidentally, similar issues of left or right-handedness were to be found when Pauling presented his α-helix models of proteins. In fact, almost all protein helices exhibit right-handedness [9]. A partial answer to that question is actually given in what is called the *full version *[10] of the preliminary article [7] (published as it happens by the Royal Society). Here we are told the following:

• that both chains follow right handed helices ...

• because left handed helices can only be constructed by violating permissible van der Waals contacts.

• We are informed that such permissible contacts include the approach of any two hydrogen atoms in the molecule to a distance of **no less **than 2.1Å.

• We are not however informed what the violations might be in a left handed helix that excludes this model. In other words, just how close can two hydrogen atoms separated (for intramolecular contacts) by at least four bonds approach? In fact, distances of ~1.85Å or less have been observed [11].

In this same full article by Watson and Crick [10], we are given a table of numerical (polar) coordinates describing the positions of twelve key atoms, but it would have taken a very determined scientist to have used only this combination of information to easily confirm the assertion that a left-handed helix is excluded. Perhaps the lack of a model with which the reader could experiment might account for the relatively slow recognition of the importance of this article in the immediate years following its publication, and the observation that whilst a physical model of DNA had of course been built, it was only available for viewing (but not modifying) by visiting Cambridge!

**Figure 3:**
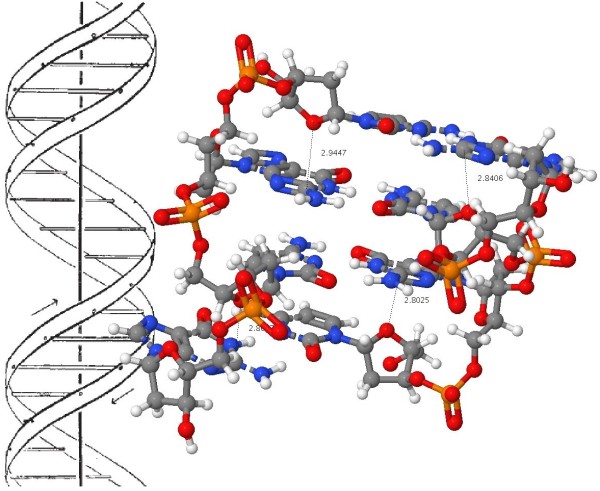
**A model of the Z-d(CGCG)_2_ DNA duplex with a geometry optimized at the ωB97XD/6-311G(d, p) level and embedded in a continuum solvent field for water**. (a) Load  coordinates for Z-d(CGCG)2 and (b) measure for close van der Waals contacts or (c) O...C contacts. (d) Load  coordinates for the diastereomeric B-d(CGCG)2 and (e) view the O...H-N and C-H...O close contacts. (f) Load Z-d(ATAT)2 and (g) view the close O...C contacts. (h) Load B-d(ATAT)2 and (i) view the close O...H-N contacts.

One tool that modern chemistry now has at its disposal (which Watson and Crick did not have) are accurate molecular models based on quantum mechanical calculations. Such a molecule is quite a challenge to model, since the computation has to take into account subtle interactions such as dispersion (long range correlation) effects, which are more or less equivalent to the van der Waals contacts referred to by Watson and Crick, the ionic phosphate groups, the planar bases and how they stack, so-called anomeric effects at the base-sugar connecting C-N bond, hydrogen bonds between both the obvious NH...N and NH...O atoms and less obvious ones such as C-H...O, and not least the capacity to deal self-consistently and accurately with the optimal positions of (at least) 250-254 atoms. In reality, such models have only very recently become available [12]. To illustrate how this famous article from 1953 [2] could now be published in a journal in 2011, I have taken the liberty of updating the original diagram with the one shown in Figure 3 (see additional file 1 for enhanced version) [6]. The additional information is made available *via *the figure caption and in Table 1 to conform to established practice in more conventional articles.

**Table 1:** Relative thermodynamic energies (kcal mol^-^^1^)^a^

System	Total energy (duplex)	Dispersion contribution	ΔΔH_298_	Δ(-T. ΔS_298_)	ΔΔG_298_ duplex	ΔG_298_ single chain	ΔΔG_298_^b^
Z-CGCG	0.0	0.0	0.0	0.0	0.0	0.0	-60.3

B-CGCG	6.2	-5.1	8.0	+3.9	+11.9	+3.1	-54.7

Z-ATAT	0.0	0.0	0.0	0.0	0.0	0.0	-44.9

B-ATAT	-7.6	-12.5	-7.0	+2.7	-4.3	-1.8	-45.7

This is a model of a DNA duplex tetramer, built using only the bases CGCG or ATAT in this example, with inclusion of three phosphate groups and calculated for both the left- and right-handed helical form. The first of these was the one deprecated by Watson and Crick on the basis that the model violates permissible van der Waals contacts. The geometry is optimized to high convergence using a recent density functional formalism (ωB97XD) [13] which incorporates a correction for the attractive dispersion component of the van der Waals interactions. Justification for the use of this functional in describing hydrogen bonding has recently been published [14]. A 6-311G(d,p) basis set results in the wavefunction being described by up to 3468 basis functions, close to the practical limit using standard computing resources available in 2010. The ionic nature of the system, deriving from the phosphate groups, was treated using a self-consistent-reaction-field continuum solvent (water) [15,16] as implemented in the Gaussian09 package, revisions A.02 and B.01. 12. In such a model, duplex formation by combining two tri-anionic chains is nevertheless exothermic in the computed free energy (Table 1), which suggests the model is not physically unrealistic. Although in principle a full ion-pair resulting from inclusion of a solvated positive counterion (typically Na^+^ or NH_4_^+^ with additional water molecules) could also be treated using this method [17], the resulting model is too large and complex for the current available computational resources.

**Figure 4:**
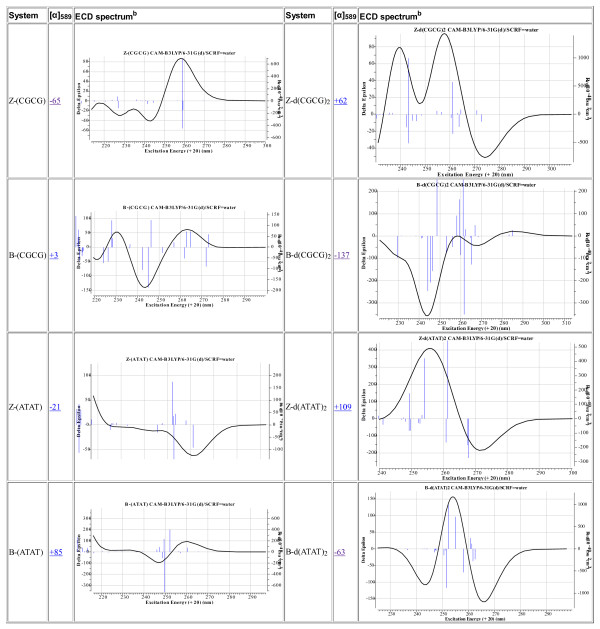
**Calculated chiro-optical properties for DNA tetramers**. ^a^Computed at geometries optimised at the ωB97XD/6-31G(d) level with with application of a SCRF solvent continuum field for water. Chiro-optical properties computed at the CAM-B3LYP/6-31G(d,p) level with application of a SCRF solvent continuum field for water. ECD spectra computed at the TD-DFT level, using Nstates = 25 and a linewidth of 0.14 with application of a SCRF solvent continuum field for water. Click on image to expand the view of the ECD spectrum. Click on expanded view of spectrum to access the digital repository entry for that spectrum. ^b^ECD spectra are presented as scalable-vector-graphical diagrams (SVG). To view, use an SVG-capable browser.

The resulting model is presented in this article using suitable software (Jmol in this instance [18]) which itself reads the optimized coordinates of all 250-254 atoms and renders these in suitable form for the reader. Annotation with identified close contacts between pairs of hydrogen atoms or other close contacts can be easily scripted in, and in principle a rich variety of actions and analyses can be built into the figure which are all based on a combination of the underlying data and algorithms implemented by the (Jmol) software. Importantly, the original data used for generating the model can be extracted from the model (the process is described here [19]) and can then be re-applied using alternative software which might provide further analysis, or indeed alternative technologies such as stereoscopic processing. These processes now turn the journal from merely a visual information source into an active scientific instrument. We may also speculate at this point on other forms of rendering data. Jmol was written in Java, and requires the browser to support a Java virtual environment. New generations of mobile information devices, which are primarily designed for long battery life, may not continue with this approach. Instead, one favoured alternative is to interface the browser directly to the graphical hardware using e.g. WebGL, and to implement the functionality of something like Jmol using the emerging HTML5 standard and appropriate scripts [20,21]. The native ability of a browser to provide such enhanced processing is already apparent in support for SVG, a markup language for vector graphics (examples of which are included in Figure 4 [see additional file 2 for enhanced version]).

The reader is invited to load the Z-d(CGCG)_2_ coordinates in Figure 3. The lengths of the van der Waals H...H attractions and the nucleophilic O...C attractions from the ribose ether oxygen to the electrophilic carbons on the guanine base have been enumerated. Because of the complex 3D nature of this molecule, they can only be truly perceived if the structure itself can be viewed from any desired angle (something clearly not possible in a conventional journal diagram). It also allows successive layers to be viewed, each perhaps concentrating on a particular aspect, without destroying or overwhelming the initial simple elegance of the overall concept (of a double helix). The identified ~2.8Å O...C interactions to the guanine are unique to the Z or left handed helical form, and since the sum of the vdW radii [22] of these two contact atoms is ~3.22Å, these are presumed to be (electrostatically) attractive. The alternative B-d(CGCG) _2 _stereoisomer reveals these O...C contacts are absent, being instead replaced by hydrogen bonds (~1.9-2.1Å) between the ribose ether-oxygen and the NH_2_ hydrogens of the guanine. The sum of the O and H vdW radii is ~2.64Å, which suggests these are significantly attractive hydrogen bonds. There are additional C-H...O-P hydrogen bonded contacts of ~2.1-2.2Å. A similar divergence of attractive interactions emerges for chains built of AT bases. The Z-d(ATAT)_2_ duplex has only one ~2.8Å O...C interaction to the adenine, with three others having rather longer lengths (~3.0-3.1Å). The B-d(ATAT)_2_ duplex instead displays C-H...O contacts of ~2.4-2.5Å.

These differences can be more succinctly summarized as:

1. Z-d(CGCG)_2_ is stabilized (*inter alia*) by a short contact between a carbon on the guanine and the ribose ether oxygen, of which there are four per four base pairs.

2. These contacts are replaced in B-d(CGCG)_2_ by NH hydrogen bonded contacts to the ribose ether oxygen.

3. In Z-d(ATAT)_2_, the O-contacts to the adenine are much longer, which

4. in B-d(ATAT)_2_, are replaced by short CH...O contacts.

By embedding access to accurate coordinate data within Figure 3, the reader can select whatever level of detail they desire from the diagram. Part of the origins of the relative stability the Z- and B- helical forms is not simply due to the presence (or in this case absence) of "violation of permissible van der Waals contacts", but also to several types of less common but nevertheless attractive interactions which may not have been inferred by building physical models alone. Such additional insights may in turn impact upon *e*. *g*. modeling one remarkable property of the DNA polymer, its ability to be stretched to almost twice its normal length without breaking [23].

It is of course the accumulation of these effects that determines the overall stability of the structure (Table 1). The thermodynamic quantities are computed with inclusion of thermal energies, obtained by solving the appropriate partition functions using calculated vibrational frequencies. Since these require second derivatives of energy with respect to coordinates, a smaller basis set 6-31G(d) was used for the purpose (a calculation time of ~4 days on a 12-core processor is typical). The dispersion corrections were obtained at the slightly higher 6-311G(d,p) basis set level to allow interactions to H to be modelled more realistically.

These energies reveal some surprises. Firstly, the free energy for forming the duplex from the separated chains is significantly exothermic, despite the electrostatic repulsions resulting from each chain carrying a 3- charge. For the resulting helix, the B-d(CGCG)_2_ form is 11.9 kcal/mol **less **stable in terms of total free energy than the Z-isomer, but is 4.2 kcal/mol **more **stable for the dispersion/van der Waals term, the criterion suggested by Watson and Crick as generally discriminating against the Z-form (although without a specification of the base type used for the model). The greater stability of the Z-form arises from a contribution of 3.9 from the entropy and 8.0 kcal/mol from the (zero-point energy corrected) enthalpy, which dominates the less favourable dispersion term.

The formation of a B-d(ATAT)_2_ duplex is less exothermic than that of the CG duplex. It is now favoured by 5.2 kcal/mol over the Z-isomer in terms of free energy and by 12.8 kcal/mol in terms of dispersion contributions. The assertion often made [24] that the Z-helix is favoured by CG rich oligomers and the B-helix by AT-rich forms is thus confirmed by these calculations.

^a^Thermochemistry computed at geometries optimised at the ωB97XD/6-31G(d) level with application of a SCRF solvent continuum field for water, with thermal corrections derived from computed vibrational frequencies. The dispersion corrections are computed for geometries optimized at the ωB97XD/6-311G(d, p) level with application of a SCRF solvent continuum field for water. The display coordinates are those obtained at this level. ^a^Free energy for the dimerisation of a single strand to a duplex.

Armed with optimized coordinates which include the weaker interactions between atoms one can annotate the basic models revealed in Figure 3 with other (computed) properties. For example the optical rotation [α]_589_ has the value +62° for Z-d(CGCG)_2_ and -137° for the B-diastereomer, perhaps surprisingly small values for such an apparently asymmetric molecule. Also surprisingly, the corresponding experimental measurement does not appear to have been reported. Optical rotations are known to be rather fragile, being sensitive to small variations in conformation and solvation, but the electronic circular dichroism spectrum is regarded as rather more robust. Unlike other forms of spectroscopy such as NMR or IR, which can be used to infer structure from simple rules based on the functional groups present (in other words, local properties), these chiro-optical properties tend to be more characteristic of the global features of the molecule. As a result, they can be very difficult to interpret without a reasonably accurate model based on these global properties. A quantum mechanical computation of the molecular wavefunction is one such model, and it is now increasingly routinely used to help interpret optical rotations, electronic (and vibrational) circular dichroism spectra. Theory can now handle molecules containing ~250-254 atoms, such as the DNA tetramers modelled here. Annotation of the models with the calculated ECD spectra is included here in the hope they might prove useful for the interpretation of the experimental spectra.

This example has illustrated how access to accurate data can help provide additional insights into the factors controlling the stability of molecular structures. In this case, the factors controlling the helical stability of DNA duplexes can be teased out. By incorporating these models directly into the journal article (and providing links to digital repositories where a more complete dataset can be acquired if needed) the readers of the journal have an opportunity to discover their own insights within their own spheres of interest.

## The crystal structure of 1,3-dimethylcyclobutadiene

**Figure 5:**
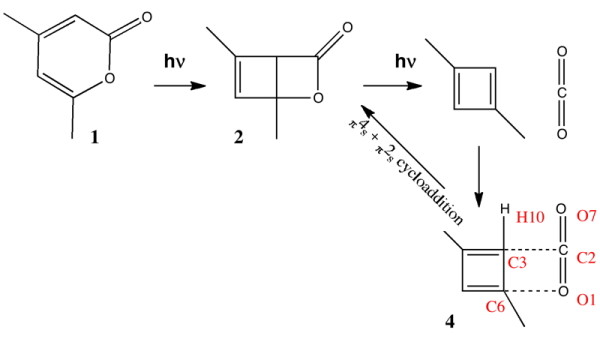
**The reaction leading to 1,3-dimethylcyclobutadiene **[25]. The numbering shown for 4 corresponds to that for the published coordinates. Load coordinates for host-guest structure and just the guest only.

The second example chosen for discussion is a more contemporary one. In July 2010, a article appeared [25] reporting the single-crystal X-ray structure of 1,3-dimethylcyclobutadiene achieved by confinement in a crystalline matrix (Figure 5 [see additional file 3 for enhanced version]). The topic caught the imagination, since cyclobutadiene has been described as the *Mona Lisa of molecules*, and its very instability means that conventional experiments on it are very challenging. The article was however conventional in the sense of being made available in (more or less equivalent) HTML and PDF versions.

Much of the scientific insight was carried in the form of four colour figures, all presented conventionally as single layered graphics with the viewpoint selected by the authors. Information on acquisition of the data on which these figures were based was given as citation 28 in that article, which lists deposition numbers (CCDC 764864-764868) and a URL that would enable a CIF file for each entry to be downloaded. Whilst this retrieval process is not entirely automatic, it does take only a few minutes to acquire the data. The usefulness of the file is of course predicated on the reader also having access to appropriate software for analysis of a file in this format. It is also important to note that a CIF file allows inspection only of the refined crystallographic model presented in the article and the statistics associated with that model; it does not allow the user access to the underlying (*hkl*) diffraction data which would allow other models to be refined and assessed.

The reaction scheme reported [25] for photochemical generation of trapped 1,3-dimethylcyclobutadiene is shown below in Figure 5. It differs from the original in showing a thermally activated reaction arrow connecting the 1,3-dimethylcyclobutadiene **4 **to **2**. This last possibility is not explicitly discussed in the original report [25], although there is there an implicit assumption that this process is slow at the temperature of the experiment, 175K. The original article therefore seeks to persuade the reader on the basis of crystallographic evidence that the structure of **3 **or **4 **has been established, with the aid of the four colour figures included in the article, and (optionally for the reader) with acquisition of the CIF files.

The theme of the present article is to ask how a reader's experience and perception of a scientific article might be enhanced or simply altered by adopting new forms of presentation. In Figure 5, the relevant CIF file can be loaded as a second layer into the reaction diagram. Because of the relatively large number of host atoms involved, the effect can be somewhat overwhelming when this is done and the interpretation may also be made more complex by the presence of disorder in the guest. A further layer of interpretation can be added by annotating the diagram with selected atom-atom distances; the reader can use the display software to added further such annotations of their own if they wish. There are many other actions the reader can perform at this point [19]. A further, this time smaller, alternative layer that contains only the kernel of the scientific problem (as perceived by the present author, which may or may not correspond to the perception of the original [25] authors) has been added here, and again four key measurement annotations made, together with selected bonds highlighted in a different colour.

The scientific problem can now be stated in the form of the following questions.

1. What are the kinetics of the reverse reaction of **4 **to give **2 **at 175K?

2. Does the crystallographic evidence convince that the guest is best described as 1,3-dimethylcyclobutadiene in close proximity to a detached molecule of carbon dioxide?

3. More specifically, how should the interaction between the labelled atoms C2 and C3 be interpreted? Should it be considered a strong van der Waals contact, as suggested by the original authors [25] or as a covalent bond? The same question might apply to another atom pair, O1 and C6 also connecting carbon dioxide and the cyclobutadiene.

4. Likewise, how should the angles O1-C2-O7 or C2-C3-H10 be interpreted?

The reader may note a common theme emerging between these questions and the origins of helical stability in DNA as discussed above.

The first of these questions was in fact posed in the form of a blog, written by the present author [26] and based on chemical precedent and entropic arguments. It was posted in August 2010, little more than a month after the original report was first published. The precedent for this form of discourse when addressing a scientific issue had already been established [27,28]. Questions 2-4 emerged more conventionally and a little later in November 2010 in the same journal as the original article, and took the form of comments submitted by two independent groups [29,30]. The original authors have a right of reply to such comments, which they took [31]. These various participants in the debate all had access to the same CIF data as is transcluded into Figure 5. The debate to this point was summarized in a second blog post [32], and this and the original post themselves attracted ~15 responses in the form of appended comments. These posed further questions, on themes such as the computed structure of 1,3-dimethylcyclobutadiene, a debate on how much energy was required for angular distortion of O1=C2=O7 as an isolated molecule, and whether molecule **2 **is transparent to light in the 320 to 500nm excitation range employed by the original experiments. This latter point was followed up by calculations of the UV-visible absorption spectrum of **2 **inside the host cavity, also appended to the blog, and finally by calculations of the predicted vibrational spectra. The next stage in the discourse occurred in a conventional journal [12], taking the form of a set of calculations on the likely barrier preventing **4 **and carbon dioxide from recombining inside the host cavity, and addressing question 1 above in more complete detail. This article did have one less conventional aspect; in the "rich HTML" version, an interactive version of the table of data was made available [33] in very much the manner adopted for Figures 3 and 5 in the present article. Additionally, there were links in this table to digital repository entries [34], which would enable any interested reader to access the complete archived details of all the calculations reported in that article.

Shortly after this last article was published, in December 2010, several publishers chose to highlight this emerging debate with editorial blog posts of their own [35-38]. These posts in turn attracted further comments, including several by one of the original authors. One comment in particular [39] entitled "Request of calculated structure data" highlighted an important aspect concerning the accessibility of previously reported data [12]. This alludes to the "rich HTML" table, and the observation that it is important to provide information on the file formats in which data is held, so that appropriate conversions if needed and concomitant visualization can be performed. This particular query was answered in the form of another blog post [19], and applies directly to the issue of how to re-use data associated with the current article (Figures 3 and 5).

The scientific discourse described above regarding the nature of the species in a host crystal lattice is still ongoing, and so a final consensus (if ever achieved) cannot be reported at the time of writing. It is noteworthy that the primary (*hkl*) crystallographic data relating to the original measurements has been provided upon request [40] and so further analysis of alternative crystallographic refinement models is now possible.

## Conclusions

The two scientific examples discussed in this article span 57 years, a relatively short period in the history of the scientific journal. The first is arguably the most influential scientific article of the 20th century, and clearly the absence of data associated with it has not held back its recognition as such. What is also clear is that addition of such data, albeit 57 years after the original report, may have the potential to reveal further insights into the structure of DNA that may not have hitherto been highlighted. Whether such a data-rich reformulation of the original problem has any measure of impact remains to be established. The second article is only months old, but in that brief period has been subjected to the kind of scrutiny that can only be achieved by having access to rich data sets. One might fairly conclude that the scientific article has evolved to enable that scrutiny. The article that you are now reading I suggest is one model for how such scientific discourse can be both improved and accelerated. It remains to be seen if scientists are prepared to author such articles in the future. There is an early example [41] of an article where both the discourse and the data supporting that discussion were seamlessly integrated into one (XML-based) document, with the presentation being made available to the reader by application of suitable stylesheet-based transformations. The production of an article in this form was however non trivial. Since then tools have appeared to facilitate the process [42,43] and the task now much be to reach both the hearts and the minds of scientific authors to encourage them to start adopting this form of enhanced scientific discourse.

## Competing interests

The author declares that they have no competing interests.

## Supplementary Material

Additional file 1 **Interactive Jmol-enhanced version of Figure **3. Click here for file

Additional file 2 **Enhanced version of Figure **4**containing additional hyper links** (This figure should be viewed with a web browser capable of SVG display, such as  Chrome,  FireFox, Safari or  IE 9).
Please note: The figure is not currently displayed as intended by the author due to technical issues with the BMC site. This will be resolved as soon as possible. Click here for file

Additional file 3 **Interactive Jmol-enhanced version of Figure **5. Click here for file
