# Supplementary material for: The past, present and future of Scientific discourse
Source: J Cheminform. 2011 Oct 14;3:46. doi: 10.1186/1758-2946-3-46 (PMC3208583; doi:10.1186/1758-2946-3-46)
Supplement: Additional file 1 — Interactive Jmol-enhanced version of Figure 3. [file 1758-2946-3-46-S1.zip › Additional file 1/index.html]

The past, present and future of Scientific Discourse


**Additional file 1:** A model of the Z-d(CGCG)2 DNA duplex with a geometry optimized at the ωB97XD/6-311G(d,p) level and embedded in a continuum solvent field for water. (a) Load coordinates for and (b) measure for close contacts or (c) contacts. (d) Load coordinates for the diastereomeric and (e) view the close contacts. (f) Load and (g) view the close contacts. (h) Load and (i) view the close contacts.

---
