# Supplementary figures and images for: The past, present and future of Scientific discourse
Source: J Cheminform. 2011 Oct 14;3:46. doi: 10.1186/1758-2946-3-46 (PMC3208583; doi:10.1186/1758-2946-3-46)

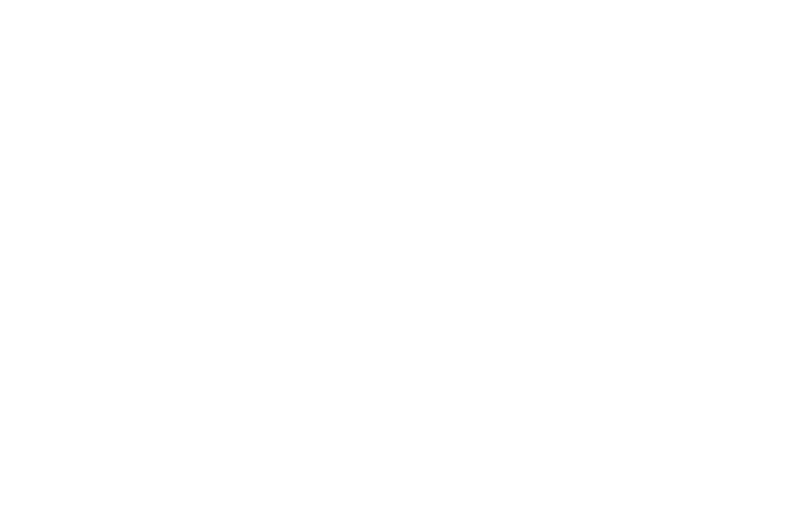

Supplement: Additional file 1 — Interactive Jmol-enhanced version of Figure 3. [file 1758-2946-3-46-S1.zip › Additional file 1/blank.jpg]

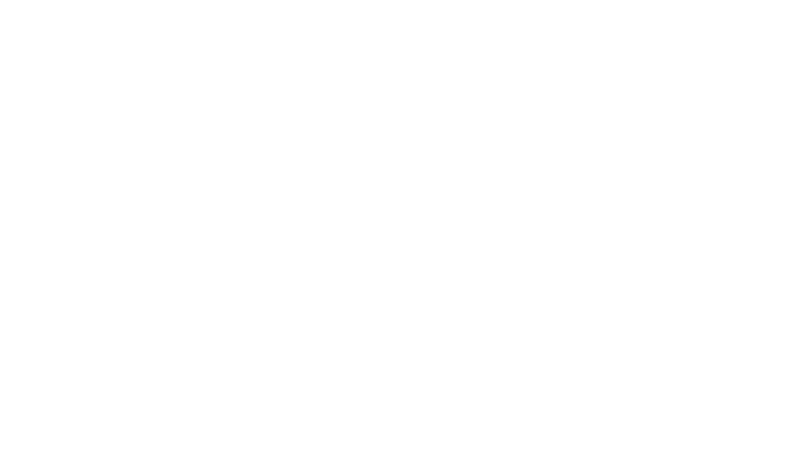

Supplement: Additional file 1 — Interactive Jmol-enhanced version of Figure 3. [file 1758-2946-3-46-S1.zip › Additional file 1/cbd-800-blank.jpg]

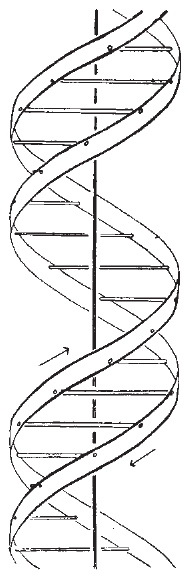

Supplement: Additional file 1 — Interactive Jmol-enhanced version of Figure 3. [file 1758-2946-3-46-S1.zip › Additional file 1/dna-helix.jpg]

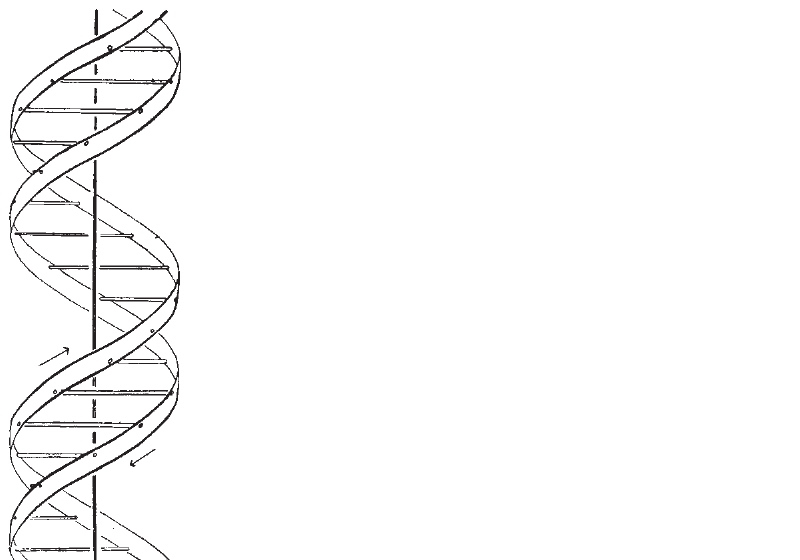

Supplement: Additional file 1 — Interactive Jmol-enhanced version of Figure 3. [file 1758-2946-3-46-S1.zip › Additional file 1/helix-back.jpg]

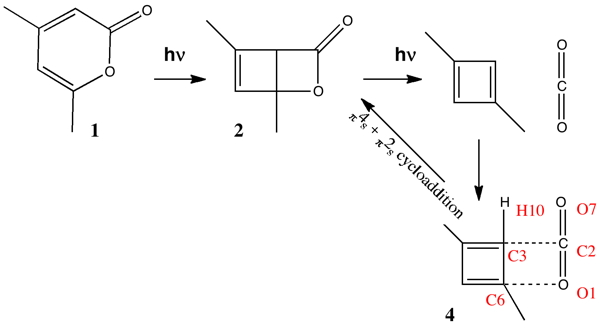

Supplement: Additional file 3 — Interactive Jmol-enhanced version of Figure 5. [file 1758-2946-3-46-S3.zip › Additional file 3/cbd-600.jpg]

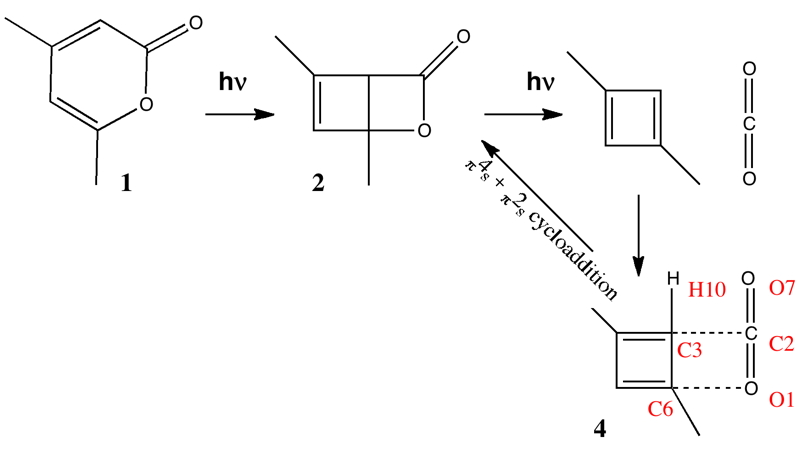

Supplement: Additional file 3 — Interactive Jmol-enhanced version of Figure 5. [file 1758-2946-3-46-S3.zip › Additional file 3/cbd-800.jpg]

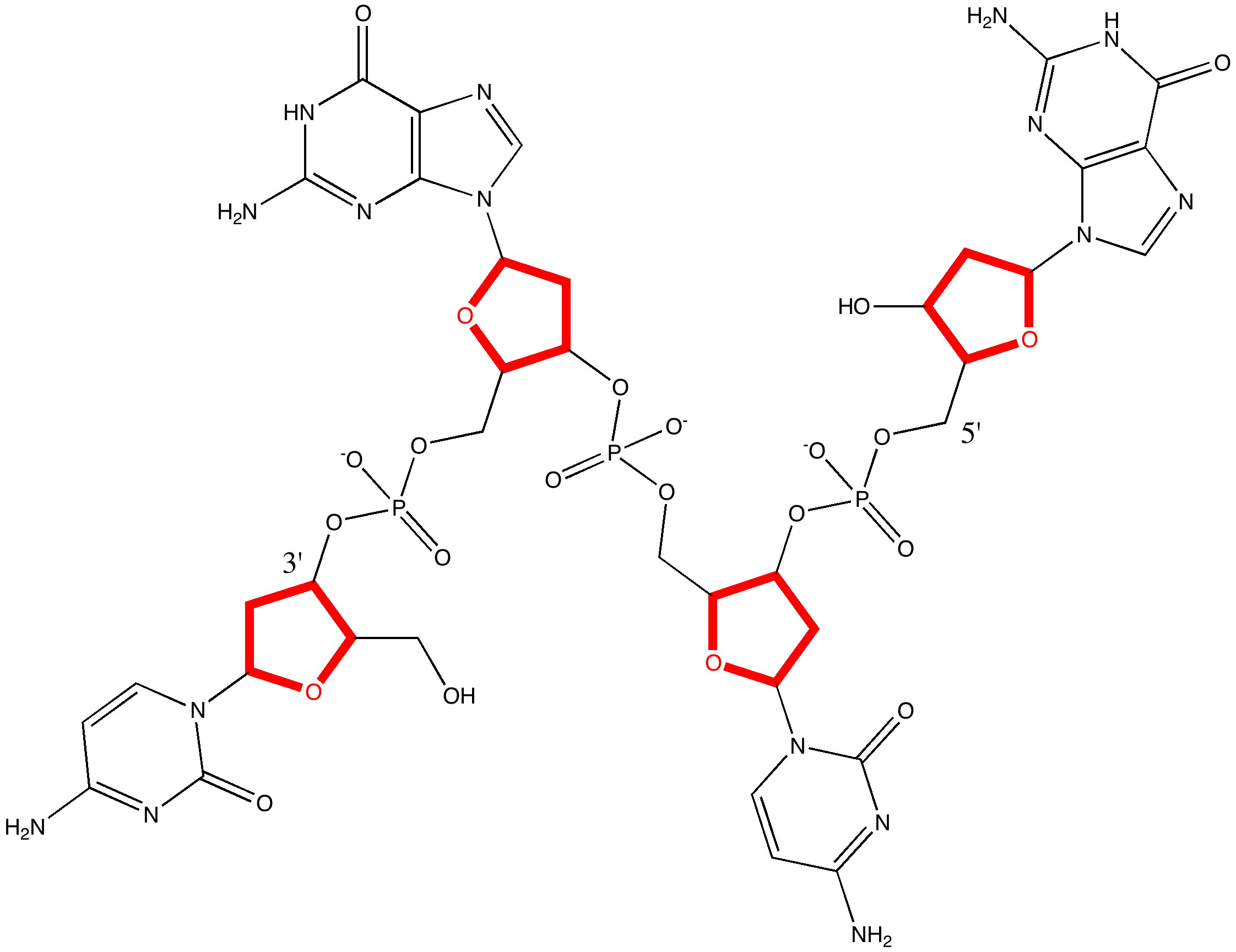

Supplement: Additional file 3 — Interactive Jmol-enhanced version of Figure 5. [file 1758-2946-3-46-S3.zip › Additional file 3/CGCG.jpg]

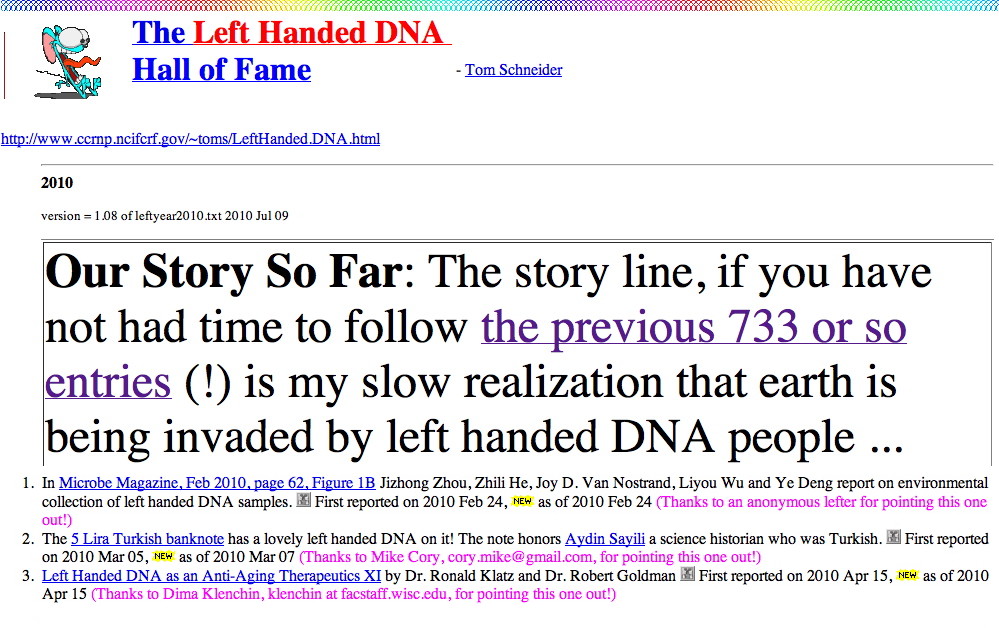

Supplement: Additional file 3 — Interactive Jmol-enhanced version of Figure 5. [file 1758-2946-3-46-S3.zip › Additional file 3/dna-left.jpg]
