# Supplementary material for: The past, present and future of Scientific discourse
Source: J Cheminform. 2011 Oct 14;3:46. doi: 10.1186/1758-2946-3-46 (PMC3208583; doi:10.1186/1758-2946-3-46)
Supplement: Additional file 2 — Enhanced version of Figure 4containing additional hyper links (This figure should be viewed with a web browser capable of SVG display, such as Chrome, FireFox, Safari or IE 9). Please note: The figure is not currently displayed as intended by the author due to technical issues with the BMC site. This will be resolved as soon as possible. [file 1758-2946-3-46-S2.zip › Additional file 2/index.html]

The past, present and future of Scientific Discourse


| Additional file 2. Calculated chiro-optical properties for DNA tetramers. | | | | | |
| --- | --- | --- | --- | --- | --- |
|  |  |  |  |  |  |
| --- | --- | --- | --- | --- | --- |
| aComputed at geometries optimised at the ωB97XD/6-31G(d) level with with application of a SCRF solvent continuum field for water. Chiro-optical properties computed at the CAM-B3LYP/6-31G(d,p) level with application of a SCRF solvent continuum field for water. ECD spectra computed at the TD-DFT level, using Nstates=25 and a linewidth of 0.14 with application of a SCRF solvent continuum field for water. Click on image to expand the view of the ECD spectrum. Click on expanded view of spectrum to access the digital repository entry for that spectrum. bECD spectra are presented as scalable-vector-graphical diagrams (SVG). To view, use an SVG-capable browser. | | | | | |
| System | [α]589 | ECD spectrumb | System | [α]589 | ECD spectrumb |
| --- | --- | --- | --- | --- | --- |
| Z-(CGCG) | -65 |  | Z-d(CGCG)2 | +62 |  |
| B-(CGCG) | +3 |  | B-d(CGCG)2 | -137 |  |
| Z-(ATAT) | -21 |  | Z-d(ATAT)2 | +109 |  |
| B-(ATAT) | +85 |  | B-d(ATAT)2 | -63 |  |
