# Supplementary material for: The past, present and future of Scientific discourse
Source: J Cheminform. 2011 Oct 14;3:46. doi: 10.1186/1758-2946-3-46 (PMC3208583; doi:10.1186/1758-2946-3-46)
Supplement: Additional file 3 — Interactive Jmol-enhanced version of Figure 5. [file 1758-2946-3-46-S3.zip › Additional file 3/index.html]

The past, present and future of Scientific Discourse


**Additional file 3:** The reaction leading to 1,3-dimethylcyclobutadiene.21 The numbering shown for **4** corresponds to that for the published coordinates. Load coordinates for and just the .

---
